# Cu-Al-Ni Nanocrystalline Compacts Obtained by Spark Plasma Sintering of Mechanically Alloyed Powders

**DOI:** 10.3390/ma17194847

**Published:** 2024-10-01

**Authors:** Calin-Virgiliu Prica, Traian Florin Marinca, Florin Popa, Argentina Niculina Sechel, Bogdan Viorel Neamțu, Horea Florin Chicinaș, Ionel Chicinaș

**Affiliations:** 1 Department of Materials Science and Engineering, Technical University of Cluj-Napoca, Muncii Ave. 103-105, 400641 Cluj-Napoca, Romania; traian.marinca@stm.utcluj.ro (T.F.M.); florin.popa@stm.utcluj.ro (F.P.); niculina.sechel@stm.utcluj.ro (A.N.S.); bogdan.neamtu@stm.utcluj.ro (B.V.N.); ionel.chicinas@stm.utcluj.ro (I.C.); 2 SC Gühring SRL, 32 Constructorilor Street, 407035 Apahida, Romania; horea.chicinas@guehring.de

**Keywords:** Cu base alloy, mechanical alloying, spark plasma sintering

## Abstract

The aim of this work is to obtain Cu-13.5Al-4Ni alloy for use as shape memory alloy by Spark Plasma Sintering (SPS) of mechanically alloyed powder. The study investigates the structural and microstructural changes in terms of crystal parameters, crystallite sizes, and phases evolution during mechanical alloying and spark plasma sintering of Cu-13.5Al-4Ni powders. We obtained alloyed powders with a structure composed of α(Cu), AlNi intermetallic compound and small amounts of elemental Al through the mechanical alloying technique. After spark plasma sintering at 900 °C, the microstructure consists of an AlNi compound distributed at the edge of α(Cu) grains. The crystallite sizes of both, α(Cu) and AlNi are in nanoscale order after 16 h of milling (9 and 6.5 nm respectively). After sintering at 900 °C (in Ar atmosphere, without holding time), the crystallite sizes increase to 46 nm for α(Cu) and to 40 nm for AlNi compound. Also, the Cu-13.5Al-4Ni compacts achieve a final density after sintering at 900 °C of around 80% from the theoretical density.

## 1. Introduction

Cu-Al-Ni alloys are known for their good mechanical properties (strength, fatigue life, and toughness), high corrosion resistance (especially in seawater), and excellent wear. As a result of these properties, they have the following applications: bearing, gears, ship propellers, and valves that handle sea water [1,2].

The Cu-Al-Ni alloys with more than 12% Al, are part of the shape memory alloys (SMA) [3,4]. The main copper-based shape memory alloys (which have come into commercial use) are the Cu-Al-Ni and Cu-Zn-Al types [5]. Cu-Al-Ni alloys are preferred as they have higher thermal stability than Cu-Zn-Al alloys. Therefore, Cu-Al-Ni alloys are used in temperature applications (around 200 °C). For this reason, they can be included in high-temperature shape memory alloys (HTSMAs) category [6]. Cu-Al-Ni alloys have become interesting recently mainly due to their low cost of obtaining compared to other alloys in this category such as Ti–Ta, Ta–Ru, Ni–Mn–Ga and Ti–Ni–Pd HTSMAs [7,8]. Among the most widely used methods of obtaining Cu-Al-Ni SMA alloys are melting and casting in an induction furnace under a protective atmosphere [9,10]. However, the low cost of production by classical methods (melting and casting) also brings some disadvantages: large crystallite sizes and/or intergranular cracking [11,12]. These shortcomings can be compensated by unconventional methods of obtaining and processing the Cu-Al-Ni alloys in order to increase the plasticity and to crystallite refinement. One of these methods consists of adding some alloying elements such as Co, Ti, Zr, Ag, Mn or Si [13,14,15,16]. Also, the powder metallurgy can be suitable for Cu-Al-Ni SMA alloys obtained due to the ability to obtain structures with fine crystallites, even in the nanometric range [17]. Mechanical alloying (MA) can be a suitable method for the synthesis of Cu-Al-Ni alloys. Mechanical alloying is a far-equilibrium technique involving the synthesizing of materials by high-energy ball milling of a powder mixture. This technique is used to obtain non-equilibrium structures, including amorphous alloys, metastable crystalline phases and nanosized materials with extended solubility and intermetallic compounds [18]. SMA of the Cu-Al-Ni and Cu-Al-Ni-Mn types were obtained by mechanical alloying starting from elemental powders up to 40 h of milling under an inert atmosphere [5,19,20]. Also, the Cu-Al-Nb SMA alloyed powders were obtained by MA with Zn stearate and MoS_2_ lubricants after 65 h [21,22]. The mechanically alloyed powder with nanocrystallites can then be put into the desired shape by Spark Plasma Sintering (SPS). These combinate methods, specifically powder metallurgy, preserve the nanostructure obtained by mechanical alloying by spark plasma sintering [23]. Spark plasma sintering (SPS) is a novel powder consolidation technique characterized by simultaneous application of pressure and a pulsed continuous current [24]. The SPS technique has been applied recently to fabricate Cu alloys with finer grain sizes and with good strength compared to cast Cu alloys [25,26,27]. Zhai et al. has obtained SPS-ed compacts from nickel-aluminum bronze alloys developed via gas-atomization [28]. The present study is focused on the synthesis of Cu-13.5Al-4Ni nanocrystalline compacts by plasma sintering starting from mechanically alloyed powders. Also, the milling time and SPS temperature impact on the microstructure of the Cu-13.5Al-4Ni alloy in terms of crystal parameters, crystallite sizes, phase evolution, and relative density was investigated.

## 2. Experimental

In the MA process, a mixture of elemental Cu, Al and Ni powders with a purity of over 99.5% was used as raw material. The composition of the mixture is listed in Table 1. The powder particle size used in the experimental studies is as follows: −50 μm for copper and aluminum, −10 μm for nickel.

The powders were milled for up to 16 h in a Fritsch Pulverisette 6 (high-energy planetary ball mill), using a hardened steel vial and balls. The mechanical alloying process was carried out in an argon atmosphere, to prevent the sample oxidation. The-ball-to-powder mass ratio—BPR was 10:1. The vial rotation speed was set at 300 rpm. Several milling times, ranging from one to 16 h were chosen for collection of samples. The nanocrystalline compacts were obtained from the powders milled for 16 h using the spark plasma sintering (SPS) technique.

**Figure 1:**
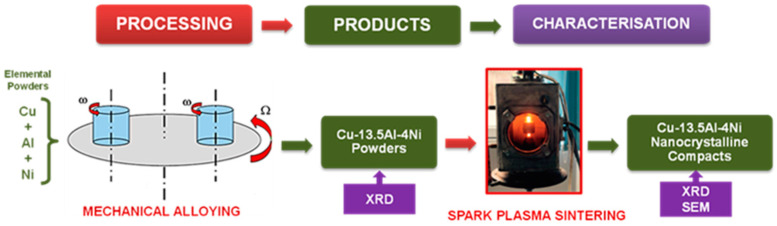
The schematic representation of the work methodology.

A home-made SPS equipment working at 24 V and 3.75 kA was used. The schematic representation of the work methodology is illustrated in Figure 1. Sintering parameters used for as-obtained sintered compacts are as follows: temperature ranging from 600 to 900 °C, and sintering time was 0 min. (without holding time), the pressure applied during sintering was 30 MPa, and the sintering atmosphere was Ar. The sintering temperature was measured with a thermocouple inserted in the graphite mold at 1 mm from the sample.

The metallographic specimens have been obtained by grinding and polishing with Struers Tegramin-30 equipment.

A JEOL-JSM 5600 LV (Tokyo, Japan) scanning electron microscope (SEM) coupled with an energy-dispersive X-ray (EDX) spectrometer UltimMAX65 (Oxford Instruments, Aztec software, version 4.2, High Wycombe, UK) was used for the investigation of particles morphology and local chemical homogeneity. For the X-ray diffraction studies, an Inel Equinox 3000 (INEL, Artenay, France) diffractometer with Co radiation (λ_Co Kα_ = 0.17903 nm) was used. The diffraction patterns were recorded in the angular range 2θ = 40–100°. The acquisition was performed in one-step in the entire two theta range, specific for INEL diffractometer. For each diffraction, the acquisition time was 10 min.

Lattice parameters were computed using the Bragg law:

(1) nλ=2dsinθ

where, λ = X-Ray wavelength (λ_Co Kα_ = 0.17903 nm), n—order of the diffraction peak, d—interplane distance and θ—diffraction angle.

From the above equation, d—interplane distance was determined. Between d and a (lattice parameter) is the following relationship:

(2) $$d=\frac{a_{0}}{\sqrt{h^{2}+k^{2}+l^{2}}}$$

h, k, l—are Miller indices determined from the indexing of the diffractograms.

The mean crystallite sizes were computed using Scherrer method.

(3) $$D=\frac{k\times \lambda }{\beta \times cos\theta }$$

where, D—mean crystallite size, k—shape factor (usually taken as about 0.9), λ = X-ray wavelength, β—line broadening in radians and θ—Bragg angle [29].

## 3. Results and Discussions

**Figure 2:**
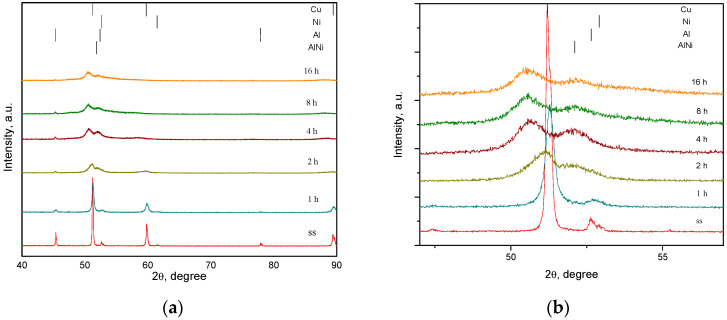
X-ray diffraction patterns of Cu-13.5Al-4Ni powders milled up 16 h (**a**) and detailed for the low angles (**b**).

The X-ray diffraction patterns of powders milled up 16 h are shown in Figure 2a. In the diffraction pattern of the starting sample (ss), only the Bragg characteristic peaks of Cu, Al, and Ni elemental powders it shows. The patterns have been indexed using the JCPDS files no. 04-0836 for Cu, 04-0787 for Al, and 04-0850 for Ni. In the first two hours of milling, the peaks of elemental powders were widening due to both, crystallite size reduction and internal stress induced in powders by the milling process. The intensities of the Ni diffraction peaks become indistinguishable after 2 h of milling. At further milling, the shift of Cu maxima to small angles takes place. This shift is highlighted in the detail presented in Figure 2b. This indicated the formation of a solid solution, the Ni atoms dissolve in the Cu lattice. It can be noticed that according to the phase diagram, the solubility between Cu and Ni in solid state is complete [30]. Also, after 2 h of milling, in the diffraction pattern, the peaks of the AlNi intermetallic compound, were identified (JCPDS files no. 44-1188). The intensity of the peaks of Al decreases with increasing milling time but can still be identified even after 16 h of milling. With increase in the milling time, up to 16 h of milling, the peaks of both, Cu base solid solution and AlNi intermetallic compound become broadened due to the internal stress of second order induced in the powders and, due to reduce the crystallite sizes.

**Figure 3:**
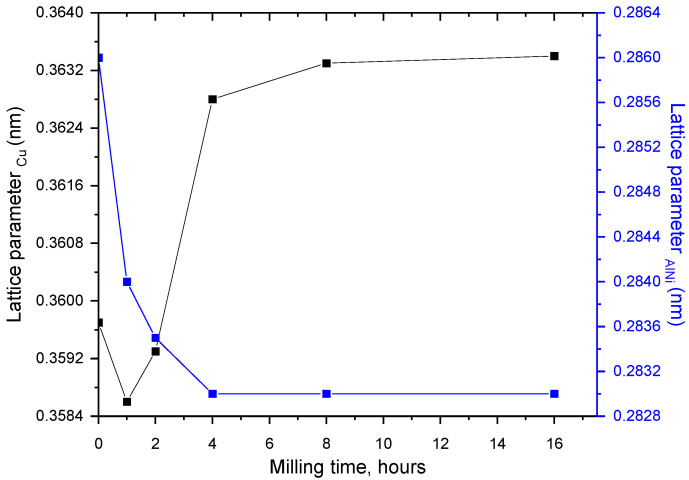
The α(Cu) and AlNi intermetallic compound lattice parameter evolution versus milling time.

The lattice parameter evolution versus milling time is shown in Figure 3. It can be observed that the lattice parameter of Cu decreases in the first hour of milling, from 0.3595 nm to 0.3585 nm. This phenomenon can be attributed to the diffusion of aluminum (atomic radius 118 pm) in the copper lattice (145 pm), leading to a shrinkage of elemental cell.

In the 1–4 h of milling range, the lattice parameter increases linearly up to a value of 0.3627 nm. This increase is due to the diffusion of Ni (atomic radius—149 pm) in Cu, resulting in a solid solution. This increase can also be attributed to the internal stresses induced by mechanical alloying/milling processes. Introducing first-order internal stresses is well known for the powder processed by this technique. With increasing milling time, from eight to 16 h, the lattice parameter reaches a saturation value, its increase being insignificant. For AlNi metallic compound, the lattice parameter decreases continuously until 4 h of milling, after which saturation is reached; in the 4–16 h range of milling, it remains constant. The explanation for this can be the diffusion of Al (small atomic radius) in Ni and change in the stoichiometry keeping in mind that the composition can vary in certain conditions, and the continuous formation of AlNi compound.

**Figure 4:**
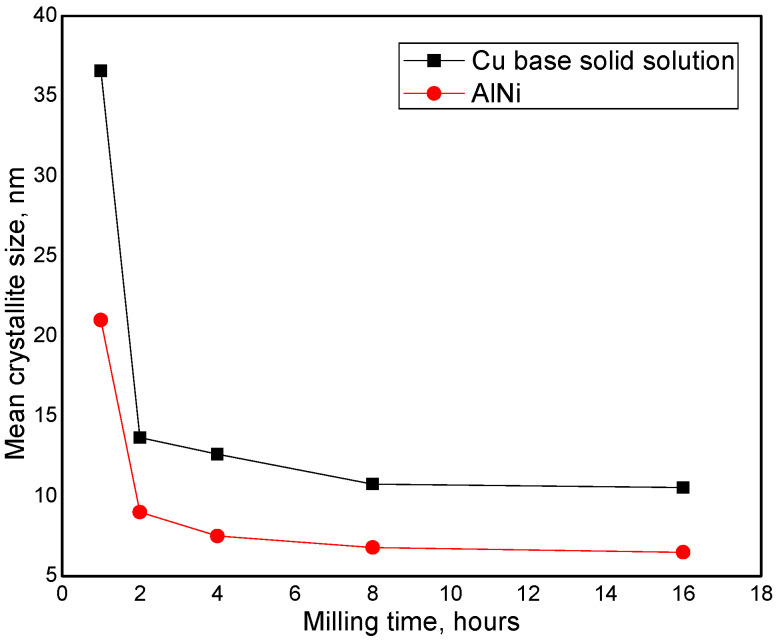
Mean crystallite sizes evolution versus milling time for α(Cu) solid solution (black line) and AlNi (red line) intermetallic compound milled up to 16 h.

Figure 4 shows the mean crystallite sizes versus milling time for Cu solid solution and AlNi intermetallic compound. The mean crystallite sizes, decrease suddenly in the first 2 h of milling. It can be observed that the crystallites of AlNi compound reached a value of 9 nm after 2 h of milling, compared to a higher value (13 nm) for Cu base solid solution. The smaller size of the crystallites in the case of the AlNi intermetallic compound can be attributed and also, to the fact that the hardness and brittleness are higher than those of Cu base solid solutions and to the fact that the AlNi nucleates in Cu-based nanocrystallites. In the range of 2–16 h of milling, the mean crystallite sizes of both, Cu base solid solution and AlNi intermetallic compound do not significantly change, reaching saturation values. So, after 16 h of milling, the mean crystallite size of Cu base solid solution is 9 nm and of AlNi compound is 6.5 nm.

**Figure 5:**
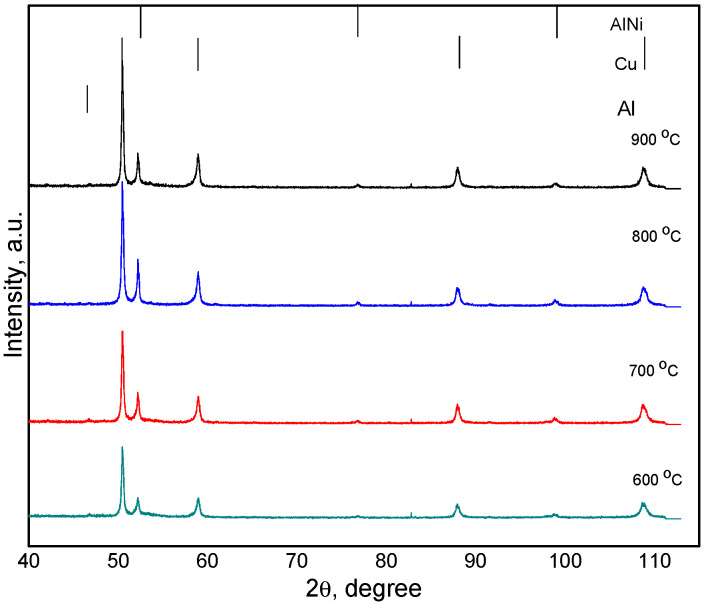
XRD diffraction patterns of Cu-13.5Al-4Ni milled for 16 h and SPS-ed compacts at 600, 700, 800 and 900 °C.

Compacts were made by Spark Plasma Sintering process from Cu-Al-Ni powders milled for 16 h milled. The XRD diffraction patterns of sintered compacts at 600, 700, 800 and 900 °C are shown in Figure 5. In diffraction patterns of sintered compacts, only the Cu base solid solution and AlNi compound were identified. With increase in the sintering temperatures, the peaks of both, Cu base solid solution and AlNi compound became narrowed due to the removal of internal stresses induced by the mechanical alloying process and the increase of crystallite sizes. The crystallite sizes increase due to recrystallization induced during spark plasma sintering by temperature increase. Also, it can be noticed that in the diffraction patterns of the sintered compacts, several diffraction maxima characteristic of the AlNi intermetallic compound appear. This can also be attributed to completing AlNi compound formation during the Spark Plasma Sintering process and the increase of crystallite size.

**Figure 6:**
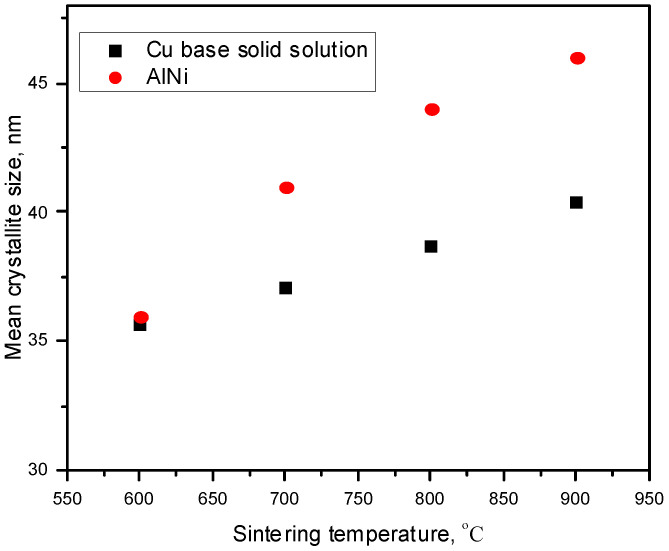
Mean crystallite sizes versus sintering temperature evolution.

In Figure 6, the mean crystallite sizes versus sintering temperature are shown. It can be observed that the crystallite sizes increase with the sintering temperature increasing (in the 600–900 °C range) for both Cu base solid solution and AlNi intermetallic compound. Thus, the mean crystallite sizes of both, Cu base solid solution and AlNi compound have approximately the same value around 36 nm when the sintering temperature is 600 °C. With the increase of the sintering temperature, the growth rate of the AlNi compound is higher than that of the Cu solid solution crystallites. So, the mean value of crystallite sizes is 46 nm for AlNi compound and 40 nm for Cu base solid solution when the sintering temperature is 900 °C.

**Figure 7:**
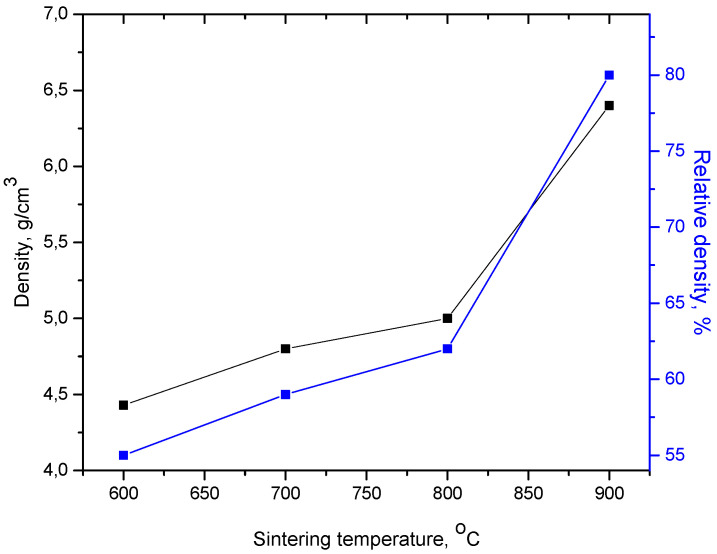
Density and relative density of the SPS-ed compacts at different sintering temperatures.

The sintered samples were weighed and dimensionally measured to determine the density after sintering. The relative density value was determined by relating the density after sintering to the value of the theoretical density of the powder mixture (8.03 g/cm^3^—according to the mixture rule). By increasing the SPS temperature, the relative density of the compacts also increases. Figure 7 shows the density and relative density of the SPS-ed compacts at different sintering temperatures. The relative density shows a moderate increase (from 55% to 62%) with the increase of the sintering temperature from 600 to 800 °C. In the 800–900 °C range of sintering temperatures, the densification of compacts is much more pronounced, with the relative density value increasing from 62% to 80%.

Morphological and compositional characterization of spark plasma sintered compacts was carried out by scanning electron microscopy (SEM) and X-ray microanalysis (EDX).

**Figure 8:**
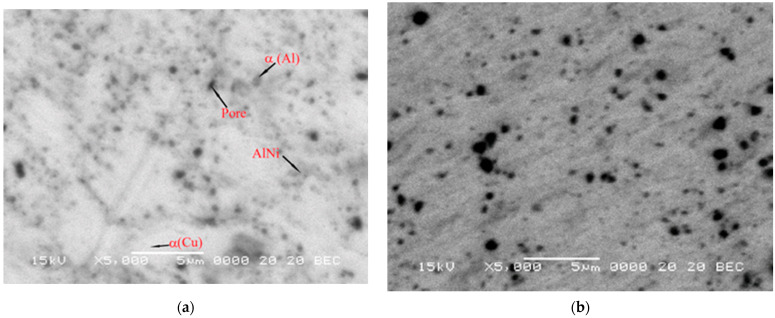

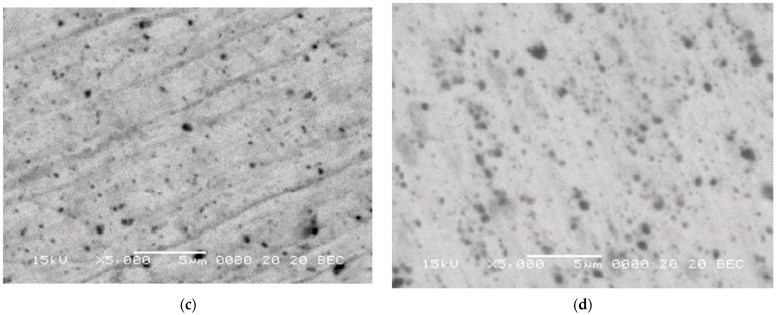
SEM images of SPS-ed compacts at 600 (**a**), 700 (**b**), 800 (**c**) and 900 °C (**d**) for the Cu-13.5Al-4Ni milled for 16 h.

Figure 8 shows the SEM image of SPS-ed compacts at 600, 700, 800 and 900 °C. SEM analyses were performed with backscattered electrons (BSE) signal. In Figure 8, the α(Al), α(Cu) and AlNi phases were identified by punctual composition analysis. By using the BSE contrast, the pores were noticed in the compacts and marked in the figure.

A similar trend can also be observed regarding the size of the pores. In addition to the pores, three areas can be identified in the microstructure: α-Cu base solid solution, AlNi intermetallic compound, and Al (Figure 8a). AlNi compound is distributed at the edge of α solid solution grains. With the increasing sintering temperature, up to 700 °C, Al areas may be identified in the microstructure (Figure 8a,b), but not at 800 °C and 900 °C (Figure 8c,d). The AlNi distribution becomes more homogeneous (Figure 8b–d). These agree with the X-ray diffraction patterns performed on the sintered compacts (Figure 5).

**Figure 9:**
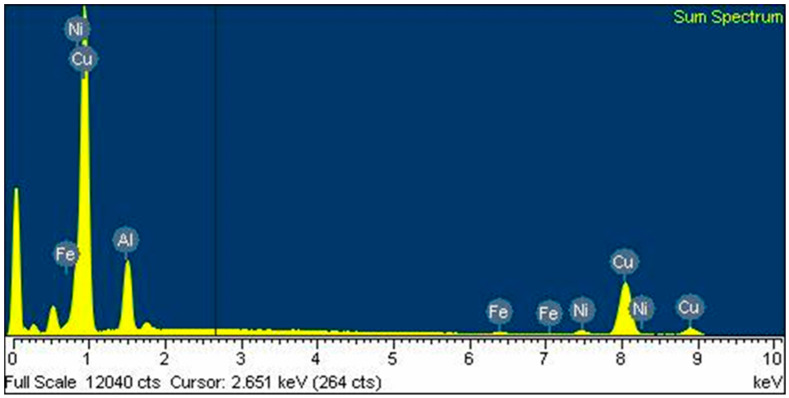
EDX spectrum of SPS-ed compact at 900 °C.

In the EDX spectrum of sintered compacts at 900 °C, besides Cu, Al, and Ni, the presence of Fe, in small quantities, is highlighted (Figure 9). The presence of 0.9 wt. % of Fe in the structure can be attributed to the contamination during the mechanical alloying process, coming from the balls and the vial.

## 4. Conclusions

The main objective of the paper was achieved; Cu-13.5Al-4Ni compacts were successfully synthesized by Spark plasma sintering of mechanically alloyed powders. Cu-13.5Al-4Ni alloyed powders were obtained from Cu, Al and Ni elemental powders by mechanical alloying technique. The 16 h-milled elemental powders of Cu, Al and Ni microstructure consist of α(Cu) solid solution, AlNi intermetallic compound and a very minimal amount of Al with mean crystallite sizes of nanometric order. After spark plasma sintering of Cu-13.5Al-4Ni alloyed powder Al disappeared from microstructure when sintering was done at 800 and 900 °C. The mean crystallite size increases from 36 nm for both, α(Cu) and AlNi compound when the sintering temperature is 600 °C to 46 nm for AlNi compound and 40 nm for α(Cu) at 900 °C of sintering temperature. The SPS process was made in an Ar atmosphere, without holding time. Also, the relative density of compacts increases with the sintering temperature increasing, reaching 80% in the case of sintering at 900 °C. The EDX analysis confirms the phase analysis provided by X-ray diffraction and shows the presence of Fe in a very small amount in SPS-ed compacts, due to the contamination during mechanical alloying.

## Figures and Tables

**Table 1:** The composition of Cu-Al-Ni powder mixture.

Powder	Composition, % wt.
Cu	82.5
Al	13.5
Ni	4

## Data Availability

Data are contained within the article.
